# Systematic Review Protocol on Antibiotic Use for infection related Orofacial Pain: Policy and Practice Implications.

**DOI:** 10.12688/f1000research.163353.2

**Published:** 2025-07-22

**Authors:** Nishant Mehta, Richa Shrivastava, Neha Patyal, Arpit Gupta

**Affiliations:** 1 Oral Health Sciences Centre, Postgraduate Institute of Medical Education and Research, Chandigarh, Chandigarh, 160012, India; 2 Oral health Sciences Centre, Postgraduate institute of Medical Education and Research, Chandigarh, Chandigarh, 160012, India; 3 Oral Health Sciences Centre, Postgraduate Institute of Medical Education and Research, Chandigarh, Chandigarh, 160012, India; 4 Oral Health Sciences Centre, Postgraduate Institute of Medical Education and Research, Chandigarh, Chandigarh, 160012, India

**Keywords:** Orofacial Pain, Antimicrobial resistance, antimicrobial, practice guidelines, clinical decision making

## Abstract

**Background:**

Dental pain and intraoral swelling, commonly resulting from pulpal and periapical infections, are leading causes of emergency visits. Overuse of antibiotics in such cases contributes to antimicrobial resistance. The appropriateness of antibiotic use must be evaluated to ensure rational prescribing and minimize unnecessary prescriptions.

**Objectives:**

This review aims to evaluate the effectiveness and associated signs and symptoms of empiric antibiotic treatment for ≤5 days compared to >5 days in patients presenting with dental pain.

**Methods:**

This protocol has been conducted following PRISMA-P guidelines. A comprehensive search of four electronic databases, such as PubMed, Scopus, Cochrane and Embase was performed to find pertinent studies. This study will follow PICOS framework, including individuals receiving antibiotic treatment for oro-facial pain. Randomized controlled trials evaluating the use of empirical antibiotics for <5 days, compared to >5 days for dental pain management. Outcomes assessed will be pain reduction, quality of life improvement, adverse events related to antibiotic, clinical resolution, microbiological and radiological findings, biomarkers, antimicrobial resistance, cost implications. Independent reviewers will screen the studies, assess the risk of bias by using RoB-2 and certainty of evidence by using GRADE. Where feasible, a meta-analysis will be conducted to synthesize findings and provide a quantitative summary of the results.

**Results:**

The study commenced in February 2025 and is anticipated to be completed by May 2025.

**Conclusions:**

This review will evaluate the effectiveness of antibiotic prescriptions for dental pain, minimizing adverse effects and identifying guideline adherence gaps. Findings will support antibiotic stewardship and inform policies to promote rational use, aiming to curb antimicrobial resistance while ensuring effective, evidence-based pain management in dental practice.

## Introduction

The global burden of dental pain significantly impacts individuals’ quality of life and can lead to costly healthcare interventions. ^1^ Dental pain and intraoral swelling are among the most common reasons why patients seek emergency care. ^2^ Dentists are among the leading antibiotic prescribers globally, contributing significantly to outpatient prescriptions. ^3^ International data indicate that dentists contribute approximately 7–10% of antibiotic prescriptions in primary care. ^4^ Antibiotics play a crucial role in managing systemic infections and preventing complications in high-risk patients, their routine use in dental pain management remains a contentious issue. ^5^ Antibiotic prophylaxis in dentistry helps prevent complications like dry sockets and infections, especially in high-risk patients. However, routine post-extraction antibiotic use is debated. Some argue that it aids healing and prevents infections, while others believe natural defenses suffice, even after surgery. ^6^ Although, While certain oro-facial infections may necessitate empirical antibiotic administration, dentists frequently prescribe antibiotics for pain management as well. ^7– 10^

The overuse and misuse of antibiotics contribute significantly to antimicrobial resistance (AMR), a growing global health crisis. ^11^ Evidence suggests that 30% to 85% of dental antibiotic prescriptions may be unnecessary or suboptimal, raising concerns about AMR and antibiotic stewardship. ^12– 14^ The World Health Organization (WHO) identifies AMR and antibiotic misuse as major global public health threats, contributing to over 6.5 million deaths annually. ^15^

Several systematic reviews and clinical guidelines outline the indications and efficacy of antibiotics for odontogenic infections. ^16– 20^ The American Dental Association (ADA) has released guidelines on the antibiotics use for managing dental pain and swellings related to endodontic infections. ^7^ These evidence-based guidelines from the American Dental Association (ADA) emphasize that antibiotics should be reserved for cases involving systemic involvement and that immediate definitive conservative dental treatment should be prioritized. ^7^ However, due to scarcity of the systematic evidence evaluating the effectiveness of antibiotics in managing oro-facial pain. Moreover, despite existing recommendations, there is no consensus on the optimal duration of antibiotic therapy for dental infections. Some studies suggest that shorter courses (≤5 days) may be as effective as longer courses (>5 days) in achieving clinical resolution while reducing the risk of resistance, adverse effects, and healthcare costs. ^21^

Moreover, several studies highlight high prescription rates among dental professionals, often driven by empirical treatment rather than clinical necessity. ^22– 26^ The inappropriate use of antibiotics for conditions that do not require them contributes to increased resistance, unnecessary side effects, financial burdens, and potential long-term health risks. ^5^Therefore, the concept of “Rational Use of Drugs” emphasizes that medications should be prescribed based on clinical necessity, effectiveness, and cost-effectiveness. ^26^ However, there is insufficient evidence in dentistry to substantiate this approach. Addressing these issues require the development of evidence-based guidelines tailored to the dental profession and improved adherence to rational prescribing practices. Given the global urgency to combat AMR and improve antibiotic prescribing practices, this review aims to assess the effectiveness of short-duration (≤5 days) versus long-duration (>5 days) antibiotic therapy in patients presenting with oro-facial pain.

## Materials

### Design and registration of protocol

The protocol follows the PRISMA-P (Preferred Reporting Items for Systematic Reviews and Meta-Analyses for Protocols) guidelines. ^27^ PRISMA-P guidelines ensure transparency and rigor in systematic review protocols by outlining key components like objectives, search strategy, and bias assessment. They enhance clarity, minimize bias, and improve reproducibility. Using PRISMA-P helps standardize reporting, facilitating peer review and evidence-based decision-making while reducing methodological deviations. Prospective registration of the study protocol was made in PROSPERO; CRD420250610118. ^28^ This systematic review will answer the following research question: ‘In patients presenting with dental pain, is empiric antibiotic treatment for less than or equal to 5 days as effective as antibiotic treatment for more than 5 days?

### Eligibility criteria

The selection of studies for this systematic review will be guided by PICOS framework (Population, Intervention, Comparison, Outcome, and Study Design), detailed as follows:

**Population:** Individuals receiving empirical antibiotic treatment for dental pain.

**Intervention:** Empirical antibiotics for a shorter duration (< 5 days).

**Comparison:** Empirical antibiotics for a longer duration (>5 days).

**Outcomes:** The primary outcomes of the study include the reduction in pain, improvement in quality of life, and assessment of adverse events associated with antibiotic use. Secondary outcomes are clinical resolution of other signs and symptoms, improvement in diagnostic parameters like microbiological tests, radiological findings, relevant biomarkers, reduction in complications, reduction in development of antibiotic resistance, cost considerations.

**Study design:** Randomized controlled trials (RCTs) will be considered eligible. Non-randomized trials, observational studies, case reports, qualitative studies, mixed-method studies, pre-post intervention studies with or without comparators, narrative/systematic reviews, and letters to the editor will be excluded.

To ensure the highest quality of evidence and reduce bias, the inclusion criteria has been limited exclusively to randomized controlled trials (RCTs), consistent with PRISMA and Cochrane guidelines. This may result in a smaller pool of eligible studies, which will be transparently reported as a limitation. In such a case, emphasis will be placed on the need for conducting future high-quality RCTs, particularly if the available trials are found to be limited in number or lacking in methodological rigor.

### Information sources and systematic search strategy

A systematic search was conducted to determine the relevant studies published in English across four electronic databases, such as PubMed, Scopus, Cochrane Library (CENTRAL) and Embase. The search strategy utilized a blend of Medical Subject Headings (MeSH) terms, primary keywords, and standardized vocabulary, integrating the following terms: ((“dental,” “teeth,” “orofacial,” “Temporomandibular,”) AND (“pain,” “ache,” “toothache,” “analges *”) AND (“antimicrobial,” “antibiotic”)). The vocabulary, search syntax, and study design filters were tailored and adapted to each database accordingly. The systematic search was conducted between February 1, 2025 and February 16, 2025 and was independently reviewed and cross-verified by the investigators. Detailed search strategies for each database are provided in Table 1. Hand search will be performed to identify relevant grey literature from Google Scholar, references of selected studies and previous systematic reviews.

**Table 1.** Search strategy.

Search engine	Search string	Results
PubMed	(“dental health services”[MeSH Terms] OR (“dental”[All Fields] AND “health”[All Fields] AND “services”[All Fields]) OR “dental health services“[All Fields] OR “dental“[All Fields] OR “dentally“[All Fields] OR “dentals“[All Fields] OR (“teeth s“[All Fields] OR “teeths“[All Fields] OR “tooth“[MeSH Terms] OR “tooth“[All Fields] OR “teeth“[All Fields] OR “tooth s“[All Fields] OR “tooths“[All Fields]) OR (“teeth s“[All Fields] OR “teeths“[All Fields] OR “tooth“[MeSH Terms] OR “tooth“[All Fields] OR “teeth“[All Fields] OR “tooth s“[All Fields] OR “tooths“[All Fields]) OR “orofacial“[All Fields] OR “Temporomandibular“[All Fields]) AND (“pain“[MeSH Terms] OR “pain“[All Fields] OR (“pain“[MeSH Terms] OR “pain“[All Fields] OR “ache“[All Fields]) OR (“toothache“[MeSH Terms] OR “toothache“[All Fields] OR “odontalgia“[All Fields] OR “odontalgias“[All Fields] OR “toothaches“[All Fields]) OR “analges*“[All Fields]) AND (“anti infective agents“[Pharmacological Action] OR “anti infective agents“[MeSH Terms] OR (“anti infective“[All Fields] AND “agents“[All Fields]) OR “anti infective agents“[All Fields] OR “antimicrobial“[All Fields] OR “antimicrobials“[All Fields] OR “antimicrobially“[All Fields] OR (“anti bacterial agents“[Pharmacological Action] OR “anti bacterial agents“[MeSH Terms] OR (“anti bacterial“[All Fields] AND “agents“[All Fields]) OR “anti bacterial agents“[All Fields] OR “antibiotic“[All Fields] OR “antibiotics“[All Fields] OR “antibiotic s“[All Fields] OR “antibiotical“[All Fields]))	2810
Embase	(antimicrobial:ti,ab,kw OR antibiotic:ti,ab,kw) AND (dental:ti,ab,kw OR tooth:ti,ab,kw OR teeth:ti,ab,kw OR orofacial:ti,ab,kw OR temporomandibular:ti,ab,kw) AND (pain:ti,ab,kw OR ache:ti,ab,kw OR toothache:ti,ab,kw OR analges*:ti,ab,kw)	1020
Scopus	((title-abs-key (dental) OR title-abs-key (tooth) OR title-abs-key (teeth) OR title-abs-key (orofacial) OR title-abs-key (temporomandibular))) AND ((title-abs-key (pain) OR title-abs-key (ache) OR title-abs-key (toothache) OR title-abs-key (analges*))) AND ((title-abs-key (antimicrobial) OR title-abs-key (antibiotic)))	4021
Cochrane Central	(dental):ti,ab,kw OR (tooth):ti,ab,kw OR (teeth):ti,ab,kw OR (orofacial):ti,ab,kw OR (temporomandibular):ti,ab,kw AND (pain):ti,ab,kw OR (ache):ti,ab,kw OR (toothache):ti,ab,kw OR (analges*):ti,ab,kw AND (antimicrobial):ti,ab,kw OR (antibiotic):ti,ab,kw	702

### Process of study selection and eligibility criteria

A reference management software, Covidence will be utilized to systematically organize all retrieved studies and eliminate duplicates. ^29^ All search results were imported into Covidence to facilitate organization, deduplication, and efficient tracking of individual studies. In first stage (Title/Abstract Screening), a team of investigators will independently screen the titles and abstracts of retrieved citations to assess their likelihood of inclusion. In second stage (Full text screening) potentially eligible articles will undergo a duplicate independent review by the investigators, who will assess them against the predefined inclusion and exclusion criteria. Any disagreements among reviewers will be clarified through group discussions to achieve a mutual agreement. The total number of identified studies will be documented in the PRISMA flow diagram.

### Process of data collection and elements

A pre-established data extraction form will be utilized by two independent reviewers to extract data from the eligible studies. The extracted data will include the author’s first name, date of publication, design of study, country of data collection, sample size, participant age range and mean, indication for antibiotic prescription, antibiotic name, route of administration, duration of use, use of additional analgesics, type of randomized controlled trial, and other key outcome measures. Any disagreements through the data collection process will be resolved through discussion between the independent reviewers. The corresponding authors will be contacted for missing or unclear data. If no response is received after two attempts, the article will be classified as having insufficient data and excluded.

### Assessment of quality of studies and certainty of evidence

The Cochrane Collaboration Risk of Bias 2 (RoB 2) tool will be used to assess the methodological quality of the included randomized controlled trials. ^30^ This tool assesses five key domains which include randomization process, deviations from intended interventions, missing outcome data, outcome measurement, and selective reporting. Study will be classified as low risk, some concerns, or high risk of bias. The results will be visually represented using a traffic light plot and a summary table to illustrate the risk of bias across studies.

The Grading of Recommendations, Assessment, Development, and Evaluation (GRADE) Framework ^31^ will be applied to determine the overall certainty of evidence. This assessment will consider risk of bias (derived from RoB 2), inconsistency (variation in results), indirectness (applicability of evidence to the research question), imprecision (confidence in effect estimates), and publication bias. Based on these factors, the evidence generated will be categorized into high, moderate, low or very low, ensuring a structured and transparent evaluation of study reliability. Any differences in data extraction will be resolved by mutual discussions. The publication bias will be assessed through funnel plots and Begg’s test if atleast 10 studies are available for meta-analysis.

***Data analysis and evidence synthesis***

*Strategy for data synthesis*

Review Manager (RevMan) Version 5.4, The Cochrane Collaboration, 2020 will be used for the meta-analysis using odds ratio and mean difference with 95% confidence interval rates. ^32^ Heterogeneity will be quantified using I ^2^ statistic test. The fixed effects model will be preferred but wherever the value of I ^2^ is more than 50% and/or clinical heterogeneity is high, the random effects model will be employed.

*Analysis of subgroups or subsets*

Subgroup analysis will be employed based on age groups (Children less than 18 yrs, Adults more than equal to 18 to 60 years, geriatric more than 60 years), disadvantaged groups (Pregnant women, lactating mothers; Patients with associated co-morbidities such as immunocompromised patients, cancer patients, diabetics, patients with liver and kidney dysfunctions etc.), severity of pain (Mild/moderate/severe), origin of pain (Endodontic origin/periodontal origin/soft tissue/glandular/bony/others) and route of administration (Oral, parenteral).

### Ethics and dissemination

Since no original data will be collected, ethical approval is not necessary. The review’s findings will be shared through publications, conference presentations, seminars, social media, and press release.

## Results

The database search is set to start in February 2025, with data extraction from individual studies scheduled for completion by March 2025. The processes of evaluating, organizing, summarizing, and analyzing the data are expected to be concluded by April 2025, while the meta-analysis and certainty of evidence assessment is projected to be finalized by May 2025.

## Discussion

Several systematic reviews and clinical guidelines emphasize antibiotic stewardship strategies aimed at minimizing unnecessary antibiotic use. ^16– 20^ This systematic review will comprehensively assess the existing evidence on antibiotic use in the management of oro-facial pain, focusing on key outcomes such as pain relief, quality of life, adverse events, AMR, clinical resolution of signs and symptoms and cost considerations. The findings of this review will provide dental practitioners and healthcare providers with an evidence-based decision-support system regarding the appropriate indications for antibiotic use, the optimal duration of therapy, and the selection of specific antibiotics for oral disease management. Additionally, the review will generate critical evidence to inform policymakers and national and international dental organizations in the development of clinical practice guidelines for antibiotic prescription and in reinforcing antimicrobial stewardship strategies within dental settings. Given the global concern surrounding antibiotic misuse, the findings of this review will also serve as a foundation for designing educational interventions aimed at promoting rational antibiotic-prescribing behaviours among dental professionals.

We anticipate potential heterogeneity among the included studies due to variations in antibiotic types, duration of use, disease conditions, and reported outcomes. To address heterogeneity, we will employ a rigorous methodological approach, including well-defined inclusion and exclusion criteria, a standardized data extraction process, and quality assessment using the Cochrane RoB 2 tool. Furthermore, subgroup analyses will be conducted based on age, disease condition, duration of antibiotic use, route of administration, and other relevant factors as needed. We expect this systematic review shall provide fresh perspectives to this field of antibiotic research in dentistry, inspiring further investigations and guiding future studies.

## Ethics and consent

Ethical approval and consent were not required.

## Data Availability

No data associated with this article. Figshare: PRISMA-P checklist for ‘[Systematic review protocol on antibiotic use for orofacial pain: Policy and practice implications]’. https://doi.org/10.6084/m9.figshare.28682657. ^33^ Data are available under the terms of the Creative Commons Zero “No rights reserved” data waiver (CC0 1.0 Public domain dedication).
