# Supplementary material for: Systematic Review Protocol on Antibiotic Use for infection related Orofacial Pain: Policy and Practice Implications
Source: F1000Res. 2025 Jul 22;14:415. Originally published 2025 Apr 8. [Version 2] doi: 10.12688/f1000research.163353.2 (PMC12334916; doi:10.12688/f1000research.163353.2)
Supplement: Peer review history

# Reviewer report: Reviewer response for version 2

**DOI:** 10.5256/f1000research.182982.r399967

**Published:** 2025-08-08

**Authors:** Sanjana Santhosh Kumar

**Affiliations:** 1 University of Rochester, Rochester, New York, USA

I did go through the revised protocol. It looks perfect and I’m fine with it

Is the study design appropriate for the research question?

Yes

Is the rationale for, and objectives of, the study clearly described?

Yes

Are sufficient details of the methods provided to allow replication by others?

Yes

Are the datasets clearly presented in a useable and accessible format?

Yes

Reviewer Expertise:

NA

I confirm that I have read this submission and believe that I have an appropriate level of expertise to confirm that it is of an acceptable scientific standard.

# Reviewer report: Reviewer response for version 1

**DOI:** 10.5256/f1000research.179686.r384236

**Published:** 2025-05-27

**Authors:** Sanjana Santhosh Kumar

**Affiliations:** 1 University of Rochester, Rochester, New York, USA

The topic is very relevant and important to acknowledge. The introduction is well written. The methods section is comprehensive; however, the rationale for including only randomized controlled trials (RCTs) warrants further justification, especially considering the potential scarcity of such studies in the context of dental antibiotic research. Additionally, the date of the hand search has not been specified. The results of the search strategy are incomplete, as they do not provide a breakdown of the number of articles retrieved from each database until the day of submission of this protocol.

Is the study design appropriate for the research question?

Yes

Is the rationale for, and objectives of, the study clearly described?

Yes

Are sufficient details of the methods provided to allow replication by others?

Yes

Are the datasets clearly presented in a useable and accessible format?

Yes

Reviewer Expertise:

Oral health, Orthodontics, Orofacial Pain

I confirm that I have read this submission and believe that I have an appropriate level of expertise to confirm that it is of an acceptable scientific standard, however I have significant reservations, as outlined above.

# Reviewer report: Reviewer response for version 1

**DOI:** 10.5256/f1000research.179686.r384234

**Published:** 2025-05-26

**Authors:** Lydia N. Melek

**Affiliations:** 1 Alexandria University, Alexandria, Egypt

The protocol is accurately designed and written following the guidelines of systematic reviews. The topic is interesting and useful for practitioners. However, I prefer adding the word "resulting from infection" to "orofacial pain" in the title. The word orofacial pain on its own can indicate concepts different from the one meant here. Wishing the authors the best of luck with their completed research.

Is the study design appropriate for the research question?

Yes

Is the rationale for, and objectives of, the study clearly described?

Yes

Are sufficient details of the methods provided to allow replication by others?

Yes

Are the datasets clearly presented in a useable and accessible format?

Not applicable

Reviewer Expertise:

Oral and Maxillofacial Surgery

I confirm that I have read this submission and believe that I have an appropriate level of expertise to confirm that it is of an acceptable scientific standard.
